# Does TSH Trigger the Anti-thyroid Autoimmune Processes? Observation on a Large Cohort of Naive Patients with Thyroid Hemiagenesis

**DOI:** 10.1007/s00005-016-0393-y

**Published:** 2016-03-14

**Authors:** Ewelina Szczepanek-Parulska, Ariadna Zybek-Kocik, Kosma Woliński, Barbara Czarnocka, Marek Ruchała

**Affiliations:** Department of Endocrinology, Metabolism and Internal Medicine, Poznan University of Medical Sciences, Przybyszewskiego 49, 60-355 Poznan, Poland; Department of Biochemistry and Molecular Biology, Center of Postgraduate Medical Education, Warsaw, Poland

**Keywords:** Thyroid, Autoimmunity, Hormone secretion, Nodular goiter

## Abstract

Thyroid hemiagenesis (THA) is a rare abnormality characterized by the absence of one thyroid lobe. Elevated thyroid stimulating hormone (TSH) level and higher incidence of thyroid diseases were reported in THA. The aim of the study is to evaluate the thyroid autoimmunity incidence in patients with THA and influence of higher than average TSH level on thyroid volume (TV) and its change with age. The study included a group of naive patients with THA and a control group of subjects with bilobate thyroid. All patients underwent clinical examination, thyroid ultrasound, scintiscan and laboratory tests. In the studied and control group the presence of thyroid autoantibodies (TAb) was evaluated. The THA group consisted of 65 patients. In THA group 53.85 % of patients were positive for TAb. Patients with positive TAb were older (46.0 ± 18.3 years) than those with negative (35.0 ± 19.8 years); *p* = 0.02. The incidence of TAb was lower in controls (13.85 %, *p* < 0.0001). In the study group, positive correlation between the age and TV (*r* = 0.46, *p* = 0.0001), and negative correlations between the age and TSH level (*r* = −0.31, *p* = 0.01), and TSH concentration and TV (*r* = −0.35, *p* = 0.004) were found. In a subgroup of 30 patients with THA negative for TAb, even stronger correlations were observed. The median single lobe volume and median TSH level were higher in patients with THA when compared to controls (13.60 vs 8.20 ml, *p* < 0.0001; 3.23 vs 1.48 µU/ml, *p* < 0.0001, respectively). Patients with THA constitute an in vivo model of long-term thyroid TSH overstimulation. Further studies are needed to reveal, whether TSH overstimulation may be the trigger for thyroid autoimmunity.

## Introduction

Thyroid hemiagenesis (THA) is a form of thyroid dysgenesis that is characterized by the absence of one thyroid lobe, with or without isthmus. Originally described in 1866 by Handfield-Jones, it is considered to be a rare, congenital anomaly (Melnick and Stemkowski 1981). To date, reported prevalence of this disorder varies 0.05–0.5 % of the population; however, the true incidence is still unknown (Duarte et al. 2009; Gursoy et al. 2008; Korpal-Szczyrska et al. 2008; Maiorana et al. 2003; Shabana et al. 2000). Since the remaining lobe is usually capable to cover hormonal requirements and sustain clinical euthyroidism, THA is most often discovered accidentally while performing different neck examinations. However, cases of overt hypothyroidism due to lack of one thyroid lobe were also reported in children by Calaciura et al. (2002) and Devos et al. (1999).

The available data about this developmental disorder are sparse. There are only few studies that analyzed large groups of affected subjects. The study published previously by our team remains the first systematic analysis of a large cohort of 40 patients with THA (Ruchala et al. 2010). It was demonstrated that mean thyroid stimulating hormone (TSH) and free triiodothyronine (FT3) values were significantly higher if compared to controls with bilobate gland. That may result in constant thyroid overstimulation with TSH and hypertrophy of the parenchyma. What is more, very often those patients were diagnosed with various concomitant thyroid diseases. Some of the most frequently observed pathologies were autoimmune thyroid disorders. However, up to now, the full evaluation of incidence and profile of thyroid autoimmunity in patients affected by THA has not been performed. The aim of the present study is to examine on a largest ever evaluated group of patients diagnosed with THA prevalence/incidence and profile of thyroid autoimmunity and possible effect of high TSH level on thyroid volume (TV) and its change with the patient age.

## Materials and Methods

The study group consisted of 65 patients (56 women and 9 men) newly diagnosed with THA, referred to our department between January 2002 and July 2013. The patients were referred because of the suspicion of thyroid disorder or THA incidentally discovered earlier during other imaging techniques by family physician, or were hospitalized in the Department of Endocrinology due to reasons unrelated to the study. The mean age was 40.9 ± 19.7 years.

The control group of 65 subjects with bilobate thyroid (56 women and 9 men), matched for age (40.3 ± 11.1 years) and gender, was chosen from participants of a population-based screening program conducted in our department.

All subjects underwent routine clinical examination, thyroid ultrasound examination, technetium scintiscan and laboratory tests, including TSH, free thyroid hormones and anti-thyroid autoantibodies (TAb) levels measurement. Additionally, fine-needle aspiration biopsy of detected focal lesions was performed, if indicated. Measurements of serum TSH, FT3 and free thyroxine (FT4) concentrations was performed using Hitachi Cobas e601 chemiluminescent analyzer (Roche Diagnostics, USA). The used reference ranges were as follows: TSH 0.27–4.2 μIU/ml, FT3 3.90–6.70 pmol/L, 27 FT4 11.5–21.0 pmol/L. Serum anti-thyroid peroxidase antibodies (TPOAb), anti-thyroglobulin antibodies (TgAb) and anti-TSH receptor antibodies (TRAb) levels were assessed by radioimmunological method with commercially available BRAMHS anti-TPO, anti-Tg, and TRAK RIA kits using scintillation gamma counter (LKB Wallac CliniGamma Counter 1272 by LKB Instruments). The cutoff of the concentration of autoantibodies, which was considered to be positive were as follows: above 2 IU/l for TRAb, above 60 IU/ml for TPOAb and 115 IU/ml for TgAb.

Ultrasonography of the thyroid was performed using an ALOKA SSD 3500 SV and AIXPLORER system by Supersonic Imagine with a probe frequency range between 7.5 and 15 MHz. All the examinations were conducted by two experienced thyroid sonographers (M R and E S-P). The size, volume and structure of the lobe were depicted. The volume has been estimated using standard formula for an ellipsoid with a correction factor 0.5. In accordance with current international guidelines, the upper limit of bilobate TV was considered as 18 ml for females and 25 ml for males. Hence, the upper limit of single lobe volume was approximately 9 and 12.5 ml, respectively.

The THA was diagnosed if the lobe absence was visualized in sonography and unilateral radionuclide uptake in the scintiscan was confirmed.

The protocol of the study was specifically approved by the Bioethical Committee of Poznan University of Medical Sciences, and all the participants gave informed written consent to participate. The research has been carried out in accordance with The Code of Ethics of the World Medical Association (Declaration of Helsinki) for experiments involving humans. The privacy rights of human subjects have always been respected.

Obtained results were statistically analyzed. All calculations were performed using Statistica 10 from StatSoft. The *p* value of less than 0.05 was considered statistically significant. Statistical significance for contingency tables was calculated using Fisher’s exact test. Significance of difference between two means was calculated by Student’s *t* test for independent samples. Presence of normal distribution of particular parameters was evaluated using the Kolmogorov–Smirnov test. Correlations were calculated using Spearman’s rank correlation coefficient test due to lack of normal distribution. Statistical difference of frequency and/or presence of evaluated factors was assessed with the use of Chi-squared test.

## Results

The studied group consisted of 65 patients, including 56 women and 9 men with a female-to-male ratio 7.6:1. The control group also enrolled 65 subjects (56 women and 9 men). The groups did not differ statistically in terms of age (*p* = 0.89).

**Fig. 1:**
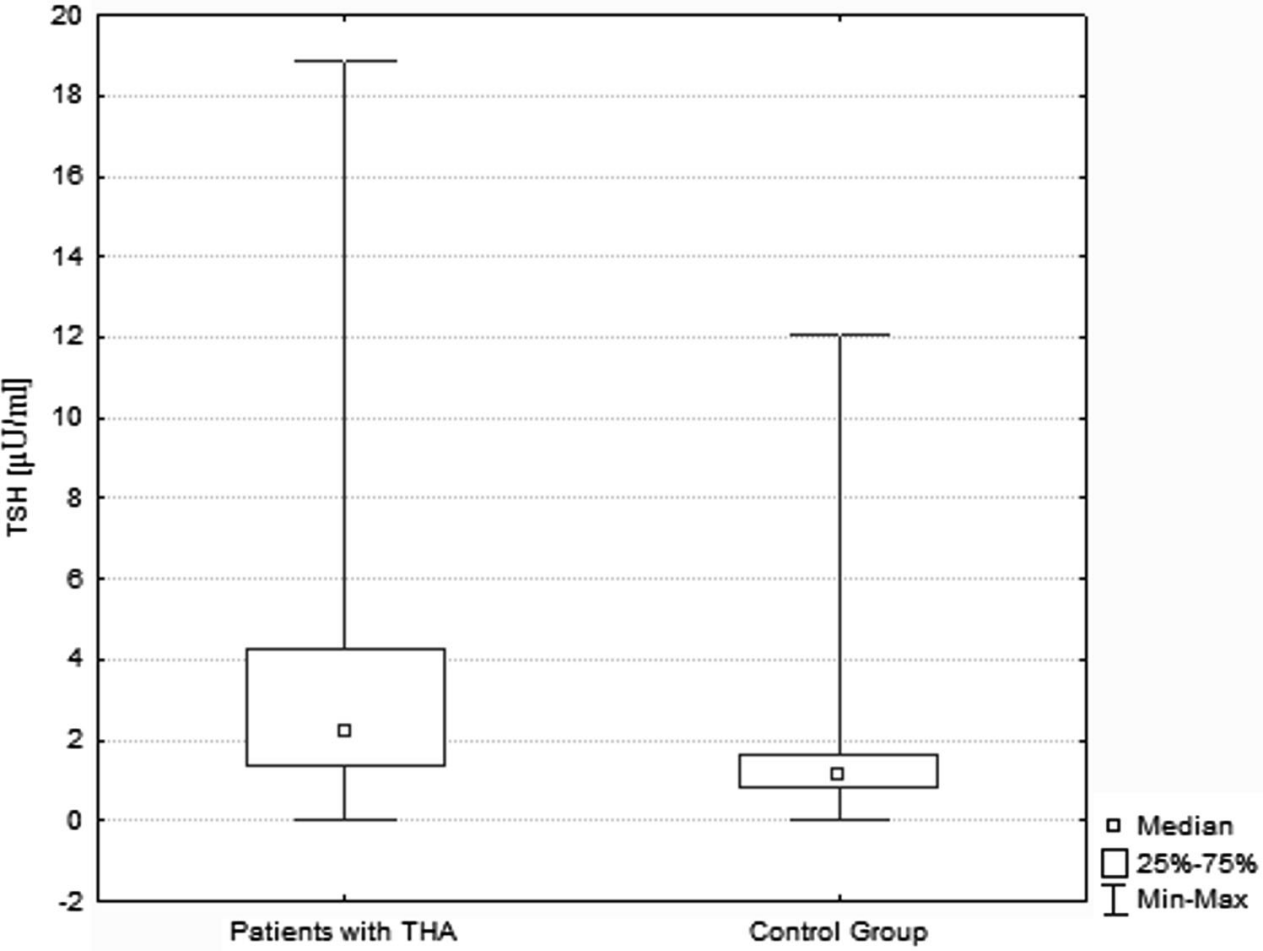
TSH level in THA and controls. The median TSH level was significantly higher in patients affected by THA (*n* = 65) if compared to bilobate controls (*n* = 65). (3.23 vs 1.48 µU/ml, *p* < 0.0001, respectively). *THA* thyroid hemiagenesis

In the study group 53.85 % (*n* = 35) of patients had elevated serum concentration of at least one type of TAb. Increased levels of TPOAb were found in 31, TgAb in 16, and TRAb in 11 cases, respectively. The patients with presence of TAb were significantly older than those without TAbs, with a mean age of 46.0 ± 18.3 and 35.0 ± 19.8 years, respectively (*p* = 0.02) (Fig. 1). The frequency of TAb detection was significantly lower (*p* < 0.0001) in the control group, where only 9 of 65 subjects (13.85 %) had increased titer of TPOAb, TgAb or TRAb. Among patients with high TAb titers, thyroid function impairment was observed in 16 cases (45.71 %); four hyper- and 12 hypothyroid patients. In the control group presence of TAb was associated with thyroid dysfunction in two cases (22.22 %). The difference was not statistically significant (*p* = 0.17).

The study group included statistically more patients with thyroid disorder than control group (22 vs 5, *p* = 0.0002). Patients from the study group were also more frequently affected by hypothyroidism than control group with no difference in frequency of hyperthyroidism (17 vs 3, *p* = 0.0007; 5 vs 2, *p* = 0.2437). Thyroid functional status was determined according to the previously described criteria (Ruchala et al. 2010). Most patients with thyroid function impairment in THA group had positive TAb (*n* = 16, 72.70 %). In the control group only two from five were positive to TAb (*n* = 2, 40.00 %). However, no statistical difference was shown between groups (*p* = 0.1611).

The majority of our patients (*n* = 28, 43.08 %) was asymptomatic on admission and THA was discovered by accident during various imaging examinations. Over one-fourth of analyzed subjects was admitted to the outpatient department because of goiter (*n* = 18, 27.69 %). The other referral indications were hyperthyroidism in 5 and hypothyroidism in 13 patients (7.69 and 20.00 %, respectively).

On ultrasound examination, heterogeneous decreased echogenicity of the thyroid parenchyma was revealed in 81.54 % of the studied patients (*n* = 53). Out of 53 patients with abnormal echogenicity, 33 were euthyroid, 15 were hypothyroid and 5 were hyperthyroid.

**Fig. 2:**
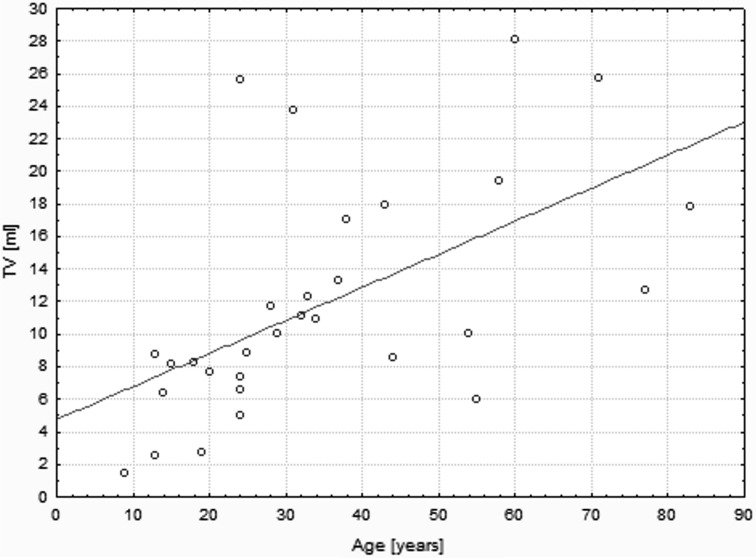
Correlation between age and TV. In the subgroup of patients with THA that did not present increased levels of TAb (*n* = 30), the significant positive correlation between the age and TV was shown (*r* = 0.68, *p* = 0.00003). *THA* thyroid hemiagenesis, *TAb* anti-thyroid autoantibodies, *TV* thyroid volume

**Fig. 3:**
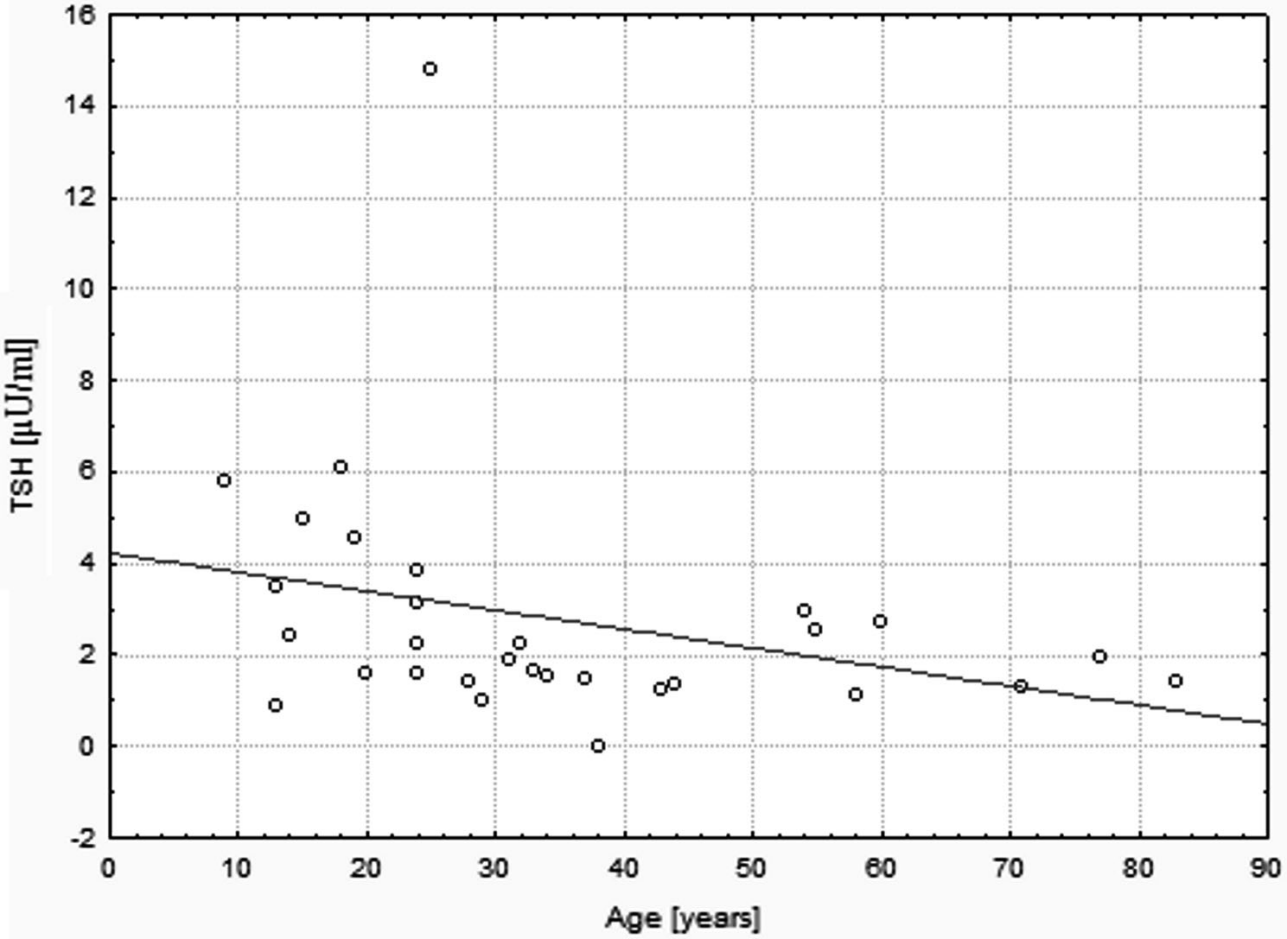
Correlation between age and TSH. In the subgroup of patients with THA that did not present increased levels of TAb (*n* = 30), the significant *negative* correlation between the age and TSH concentration was noted (*r* = −0.44, *p* = 0.014). *THA* thyroid hemiagenesis, *TAb* anti-thyroid autoantibodies, *TSH* thyroid stimulating hormone

**Fig. 4:**
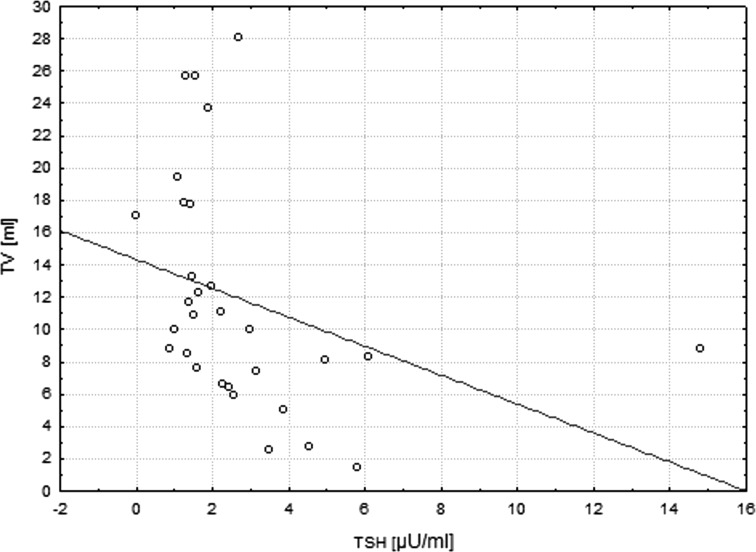
Correlation between TV and TSH. In the subgroup of patients with THA without coexisting autoimmune thyroid disease (*n* = 30), the significant *negative* correlation between the TV and TSH level was visualized (*r* = −0.58, *p* = 0.0008). *THA* thyroid hemiagenesis, *TSH* thyroid stimulating hormone, *TV* thyroid volume

The median single lobe volume as well as median TSH level were noted to be significantly higher in patients affected by THA if compared to bilobate controls (13.60 vs 8.20 ml, *p* < 0.0001; 3.23 vs 1.48 µU/ml, *p* < 0.0001, respectively) (Fig. 1). In the whole studied group a positive correlation between the age and TV of patients was demonstrated (*r* = 0.46, *p* = 0.0001). It was also shown that there was a negative correlation between the age and TSH level (*r* = −0.31, *p* = 0.01), as well as within TSH concentration and TV (*r* = −0.35, *p* = 0.004). Same analysis has been performed in the group of 30 patients, out of 65 subjects with THA that did not present increased levels of TAb. In this group similar correlations have been shown (*r* = 0.68, *p* = 0.00003; *r* = −0.44, *p* = 0.014; *r* = −0.58, *p* = 0.0008, respectively) (Figs. 2, 3, 4). No significant correlations between the age and TV, the age and TSH level and TSH concentration and TV have been demonstrated in the group of patients with detected TAb.

**Fig. 5:**
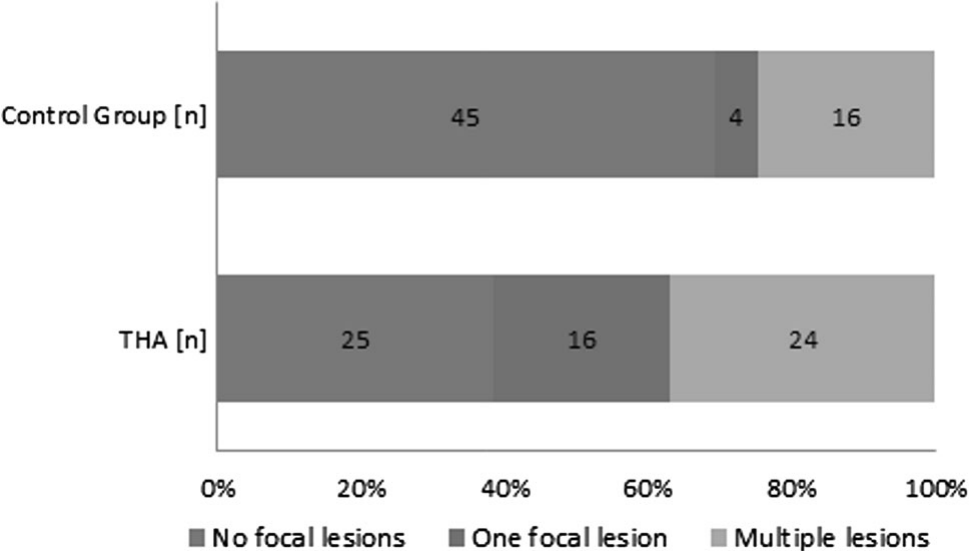
The incidence of focal lesions in THA patients (*n* = 65) and control group (*n* = 65). The distribution *chart* presents the *number* of subjects with no focal lesions, one focal lesion and multiply focal lesions of the thyroid. *THA* thyroid hemiagenesis

The incidence of focal lesions in the study group, found during thyroid ultrasound, was significantly higher 61.54 % (*n* = 40) than in the control group (*n* = 20)—30.77 % (*p* = 0.0002). In the study group 24 (60 %) and in control group 16 (80 %) subjects had more than one focal lesion (Fig. 5). In majority of cases lesions were solid nodules or cystic-solid nodules in both study and control group (*n* = 38 and *n* = 16, respectively), and only cystic lesions were present in two and four cases, respectively. The fine-needle aspiration biopsy was performed in 20 patients, and 11 of them revealed the presence of lymphocytes, suggesting chronic thyroiditis.

The results are also presented in Table 1.

**Table 1:** The values of age, single lobe volume, TSH, FT3 and FT4 as well as frequency of focal lesions, thyroid autoantibodies detection and thyroid dysfunction (hyper/hypothyroidism) in patients with thyroid hemiagenesis (THA) and control group

	THA	Control group	*p* value
Age (years)	40.89	40.27	0.8263
Single lobe volume (cm^3^)	13.60	8.20	<0.0001*
TSH (µU/ml)	3.23	1.48	<0.0001*
FT3 (pmol/L)	5.7	4.14	<0.0001*
FT4 (pmol/L)	15.49	15.62	0.8707
Focal lesions (%)	61.54 %	30.77 %	0.0002*
TAb detection (%)	53.85 %	13.85 %	<0.0001*
Hypo/hyperthyroidism (%)	33.84 %	7.69 %	0.0002*

*THA* thyroid hemiagenesis, *TAb* thyroid autoantibodies (TPOAb, TgAb, TRAb), *TSH* thyroid stimulating hormone, *FT3* free triiodothyronine, *FT4* free thyroxine

* The *p* values considered statistically significant are marked

## Discussion

Thyroid hemiagenesis remains one of the most poorly described developmental abnormalities of the thyroid gland. The origin of this anomaly is unknown. The most frequently mentioned potential causes are mutations in genes encoding thyroid transcription factors or disturbed migration and bilobation of thyroid primordium (Fagman and Nilsson 2011; Tonacchera et al. 2007). That includes for example *PAX8* and *FOXE1* gene, that have already been described to take part in the molecular background of this abnormality (Szczepanek-Parulska et al. 2013; Szczepanek et al. 2011).

To date THA has been considered to be an interesting accidental finding that is generally not clinically relevant. Most available literature about this disorder consists of case reports describing the coexistence of THA and other pathologies. Concomitant thyroid diseases included Graves’ disease (Rashid et al. 1998), Hashimoto’s thyroiditis (Lazzarin et al. 1997), postpartum silent thyroiditis (Nakamura et al. 2004), subacute thyroiditis (Shibutani et al. 1995), simple goiter (Yildiz and Bozkurt 2003), non-toxic or toxic nodular goiter (Karabay et al. 2003), thyroid cancer (Khatri et al. 1992), congenital hypothyroidism (Beltrao et al. 2010), ectopic sublingual thyroid (Yang and Hong 2008) or thyroglossal duct cyst (Tsang and Maher 1998). Some of the available case reports confirm that a number of thyroid pathologies may coexist in a single patient with THA, similarly to subjects with normally developed gland (Cakir et al. 2009; Ruchala et al. 2008). To date, some cases of concomitant parathyroid abnormalities were reported (Oruci et al. 2012). Nevertheless, in accordance with published data, the calcium phosphate balance in THA subjects is undisturbed (McHenry et al. 1995; Ruchala et al. 2011). Other extrathyroidal pathologies coexisting with THA are: familial dilated cardiomyopathy and hypergonadotrophic hypogonadism (Gursoy et al. 2006), autoimmune polyglandular syndrome type III (Papi et al. 2003), pituitary adenoma (Leiba et al. 1976), right aortic arch (Konno and Kanaya 1988), Down (Nebesio and Eugster 2009), Williams (Cammareri et al. 1999) and DiGeorge Syndrome (Fagman et al. 2007) or dysmorphic face with short stature (Vakili and Mazlouman 2003). The study conducted previously in our department, that remains the first systematic analysis in a cohort of 40 patients with THA, revealed surprisingly high incidence of autoimmune thyroid disease (AITD) in the studied group. To date, cases of THA association with AITD were described in the form of case reports. However, the selection bias might have occurred, as those patients were preferentially reported as an interesting coincidence. Cakir et al. (2009) describe the simultaneous presence of Graves’ disease, Graves’ ophthalmopathy and multinodular goiter in a 55-year-old woman. On the other hand, Bando et al. (1999) provide an interesting description of a 42-year-old female with right THA, who developed hyperthyroidism in the course of Graves’ disease after initially being hypothyroid due to Hashimoto’s thyroiditis. A similar case has been reported also by our team in 2008 (Ruchala et al. 2008). Another publication by Ammaturo et al. (2007) indicates the possible concomitance of Graves’ disease and papillary thyroid carcinoma in a patient with THA. Hypercalcemia as a result of thyrotoxicosis due to Graves’ disease has also been described in association with THA (Kebapcilar et al. 2009).

A large series of consecutive patients presenting THA has not yet been analyzed as regards the frequency of thyroid autoimmune disorders. This prompted us to investigate the issue on a large group of subjects with this disorder. The results revealed high prevalence of elevated anti-TAbs, which is significantly higher than in control subjects. Significantly more patients from the study group had clinical manifestation of thyroid autoimmune process, when most subjects from control group despite being positive for TAb had no symptoms of the disorder.

The reasons for the increase in TAb level in patients with THA are unknown. The susceptibility for AITD is mostly attributed to genetic constitution (Rapoport and McLachlan 2001; Weetman 2009). However, on the basis of some experimental studies it has been hypothesized that excessive stimulation of TSH receptors may lead to the “leak” of some thyroid autoantigens, such as thyroglobulin or thyroid peroxidase, to the circulation. That may induce an autoimmune response resulting in the TAb production (Flynn et al. 2007). However, to date there were no clinical studies addressing this issue. It is possible that prolonged overstimulation with TSH in THA subjects promotes the development of thyroid autoimmune pathologies, such as Hashimoto’s thyroiditis or Graves’ disease. Subjects with THA represent a kind of clinical model of previous experimental studies due to constant exposure to higher TSH level (Fig. 1) in response to initially reduced TV in patients with one thyroid lobe. This theory is supported by the fact, that frequency of thyroid autoimmunization was noted to rise with age.

Both experimental and clinical trials conducted so far provide evidence for great trophic influence of TSH on thyroid gland. Prolonged TSH stimulation is a well-known goitrogen and a risk factor for carcinogenesis (Boelaert 2009; Pedrinola et al. 2001). In the present study conducted on a larger group of subjects we can confirm the previous results, indicating that in patients with THA mean TSH and FT3 are significantly higher when compared to controls with bilobate thyroid gland (Ruchala et al. 2010) (Fig. 1). The results are shown in Table 1. The compensatory hypertrophy of a single lobe observed in majority of THA cases might be a direct result of increased TSH serum level and following thyroid overstimulation. We observed that there is a significant age-dependent increase in TV followed by decrease in serum TSH value (Fig. 4). The observed lobe enlargement initially probably reflects a compensatory effort: iodine storage may be impaired compared to fully developed thyroid glands and therefore synthesis of T4 is limited. Conversion of T4 to T3 is probably also limited and compensated by a relatively high T3-synthesis to sustain clinical euthyroidism. It may be hypothesized, that TSH level higher than in the control group is responsible for both thyroid parenchymal hyperplasia and thyroid focal lesions occurrence resulting in general thyroid enlargement observed in THA patients. In the group of TAb-positive patients 45.71 % had thyroid disorders which are known to significantly influence TSH and TV, interfering with the correlation between age, TSH and TV observed in patients with no thyroid function impairment (Rapoport and McLachlan 2001). The compensatory enlargement of a single lobe was also associated with largely increased number of detected focal lesions if compared to control subjects. The negative correlation between TSH and age (Fig. 3) in the groups with and without increased levels of TAb is interesting, since TSH levels were reported to increase in older patients for unknown reasons. Described findings could indicate that with increasing age accompanied by long-term TSH overstimulation some thyroid tissue might be transformed gradually into autonomously functioning tissue, often accompanied by focal lesions.

Unfortunately, our study has some limitations. The research was conducted on Polish, Caucasian population and the obtained results cannot be uncritically extrapolated to other populations. Until 1997 Poland was an iodine-deficient country, but the addition of iodine to table salt has largely eliminated this problem (Szybinski et al. 2001). However, the introduction of iodine supplementation may be associated with an increase in incidence of AITD (Baczyk et al. 2006; Fountoulakis et al. 2007; Teng et al. 2006). Thus, these findings may not be reproducible for other populations. On the other hand, the induction of thyroid autoimmunity in such populations should affect both subjects with a single and bilobate thyroid. Moreover, the studied group consisted of subjects referred to the endocrine outpatient clinic. The reasons for admission included the signs of goiter and the symptoms of hypo- and hyperthyroidism. However, this may lead to the selection bias that patients with suspicion of any thyroid disorder were preferentially referred. On the other hand, most of our patients were asymptomatic on admission and the THA diagnosis was made incidentally during the routine examination in majority of subjects. Maiorana et al. (2003) performed a systematic screening of healthy school children for thyroid developmental abnormalities, that indicated the actual higher prevalence of THA in males (male-to-female ratio 1.4:1.0), while in our study an important female preponderance was noted, like in most other published studies (Gursoy et al. 2008; Shabana et al. 2000; Shaha and Gujarati 1997). Since females are more prone to AITD (Rapoport and McLachlan 2001), that may influence the obtained data on high prevalence of thyroid autoimmunity in the studied group. Still, the incidence of AITD is much higher than in the group of control subjects, while gender proportion in both groups was identical.

In conclusion, patients with THA are a unique in vivo model of long-term thyroid overstimulation with TSH that may result in thyroid hyperplasia and the increased occurrence of goiter. What is more, obtained results support the observation that thyroid autoimmune diseases are very frequent in THA population, indicating the need for careful follow-up of subjects with THA, who are at higher risk of developing thyroid dysfunction due to limited functional reserve of unilobate thyroid and presumably increased incidence of anti-TAbs. Some further prospective studies are needed to reveal, whether constant TSH stimulation may be the trigger of AITD development. Based on the described data authors suggest considering L-thyroxine supplementation in young subjects diagnosed with THA, as a possible prevention of development of clinical pathologies potentially resulting from TSH overstimulation.
